# Bipolar disorder and diabetes mellitus: evidence for disease-modifying effects and treatment implications

**DOI:** 10.1186/s40345-016-0054-4

**Published:** 2016-07-07

**Authors:** Ellen F. Charles, Christophe G. Lambert, Berit Kerner

**Affiliations:** David Geffen School of Medicine, University of California, Los Angeles, 10833 Le Conte Ave, Los Angeles, CA 90095 USA; Center for Global Health, Division of Translational Informatics, Department of Internal Medicine, University of New Mexico Health Sciences Center, University of New Mexico, MSC10 5550, Albuquerque, NM 87131 USA; Semel Institute for Neuroscience and Human Behavior, University of California, 695 Charles E. Young Drive South, Box 951761, Los Angeles, CA 90095 USA; Fakultät für Gesundheit, Private Universität Witten/Herdecke, Alfred-Herrhausen-Straße 50, 58448 Witten, Germany

**Keywords:** Bipolar disorder, Diabetes, Epidemiology, Cohort studies, Pathophysiology, Evidence

## Abstract

**Background:**

Bipolar disorder refers to a group of chronic psychiatric disorders of mood and energy levels. While dramatic psychiatric symptoms dominate the acute phase of the diseases, the chronic course is often determined by an increasing burden of co-occurring medical conditions. High rates of diabetes mellitus in patients with bipolar disorder are particularly striking, yet unexplained. Treatment and lifestyle factors could play a significant role, and some studies also suggest shared pathophysiology and risk factors.

**Objective:**

In this systematic literature review, we explored data around the relationship between bipolar disorder and diabetes mellitus in recently published population-based cohort studies with special focus on the elderly.

**Methods:**

A systematic search in the PubMed database for the combined terms “bipolar disorder” AND “elderly” AND “diabetes” in papers published between January 2009 and December 2015 revealed 117 publications; 7 studies were large cohort studies, and therefore, were included in our review.

**Results:**

We found that age- and gender- adjusted risk for diabetes mellitus was increased in patients with bipolar disorder and vice versa (odds ratio range between 1.7 and 3.2).

**Discussion:**

Our results in large population-based cohort studies are consistent with the results of smaller studies and chart reviews. Even though it is likely that heterogeneous risk factors may play a role in diabetes mellitus and in bipolar disorder, growing evidence from cell culture experiments and animal studies suggests shared disease mechanisms. Furthermore, disease-modifying effects of bipolar disorder and diabetes mellitus on each other appear to be substantial, impacting both treatment response and outcomes.

**Conclusions:**

The risk of diabetes mellitus in patients with bipolar disorder is increased. Our findings add to the growing literature on this topic. Increasing evidence for shared disease mechanisms suggests new disease models that could explain the results of our study. A better understanding of the complex relationship between bipolar disorder and diabetes mellitus could lead to novel therapeutic approaches and improved outcomes.

## Background

Bipolar disorder (BD) refers to a group of conditions that share the defining features of elated/euphoric or irritable mood accompanied by persistently increased activity or energy levels, also known as mania (American Psychiatric Association 2013). BD occurs worldwide with a lifetime prevalence of about 0.6 % for BD-I and 0.4 % for BD-II, with slightly higher rates reported in developed countries (Merikangas et al. 2007, 2011).

Evidence for an increase in chronic medical conditions in patients with BD has been described since the pretreatment era (Esquirol 1845; Swift 1907; Rennie 1942; Stenstedt 1952; Alvarez Ariza 2009). Several disorders are frequently diagnosed in patients with BD, including epilepsy, thyroid disorders, cardiovascular diseases, autoimmune–allergic disorders, and diabetes mellitus, especially in the elderly (Lala and Sajatovic 2012; Perugi et al. 2015). Since symptoms of these somatic disorders overlap with those of BD, they could challenge the diagnostic process and delay treatment (Sajatovic and Chen 2011; Smith et al. 2013; Maina et al. 2013). Chronic medical conditions in patients with severe mental illness also lead to increased risk of frequent hospitalizations and re-hospitalizations (Davydow et al. 2015). While recent reviews of this topic have identified comorbid medical conditions in the elderly with BD as a growing public health problem (Depp and Jeste 2004; Vasudev and Thomas 2010; Dols et al. 2014; Sajatovic et al. 2015a), this patient population is often not well represented in clinical trials (Beers et al. 2014). However, case reports suggest that co-occurring medical conditions have a significant effect on the disease onset, the disease course, treatment response, and outcome (Sami et al. 2015). Diabetes mellitus appears to take center stage among these disorders.

Recent reports and one meta-analysis have suggested a relationship between BD and diabetes mellitus. However, these studies could not disentangle the effects of ethnicity, medication use and age, which could have potentially confounded the results (Vancampfort et al. 2015). Especially, the variability in the prevalence of diabetes mellitus in the background population has been rarely considered. Small sample sizes and restricted mean age range were the main limitations in most studies. In a systematic review, we have attempted to address some of these shortcomings. In contrast to previous studies, we have focused on large population-based cohort studies from diverse ethnic backgrounds with special attention to those studies that included the elderly. Then, we reviewed the evidence for shared disease mechanisms between BD and diabetes mellitus. Finally, we explored the evidence for disease-modifying effects and treatment implications.

## Methods

**Fig. 1:**
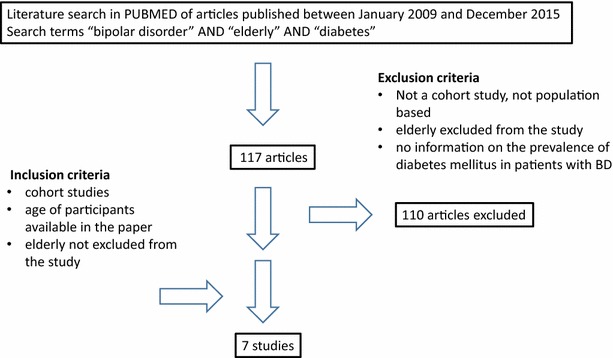
Selection process for the inclusion in the systematic review

Using the combined terms “bipolar disorder” AND “elderly” AND “diabetes”, two independent researchers have carefully searched the PubMed database for large, observational cohort studies with retrospective, cross-sectional, or prospective design published between January 2009 and December 2015. We found 117 papers; 7 studies were large cohort studies from diverse populations (Table 1), and therefore, were suitable for our review. Two reviewers independently selected the studies and extracted the data in duplicate according to predefined criteria and a study protocol that could be provided on request. Studies were included if they were population based, contain patients diagnosed with BD based on Diagnostic and Statistical Manual of Mental Disorders, 4th Edition (DSM-IV) or International Classification of Diseases (ICD) criteria, and also included patients diagnosed with diabetes mellitus. Excluded were studies that had excluded elderly patients, studies that were not population based and studies that did not mention the inclusion of patients with diabetes mellitus in addition to BD (Fig. 1). Since the number of the identified studies was too small and too diverse for meta-analysis, we refrained from a statistical analysis.

**Table 1:** Large cohort studies provide evidence for a significant association between bipolar disorder and diabetes mellitus

Author	Year	Title	Design	Type of bipolar disorder (BD)	Method of assessment of BD	Type of diabetes mellitus (DM)	Method of assessment	Results for BD group	Age of participants (years)	*N*
Wändell et al.	2014	Diabetes and psychiatric illness in the total population of Stockholm	National cohort study Cross-sectional study	BD F30–F31	Electronic patient records	DM (ICD-10 codes E10–E14	Electronic patient records	Age adjusted odds ratio of BD among patients with DM 1.714 (1.540–1.905) for women and 1.600 (1.429–1.792) for men	0–85+	2058,408 96,103 with DM 6341 with BD
Crump et al.	2013	Comorbidities and mortality in bipolar disorder: a Swedish national cohort study	National cohort study Cross-sectional	BD ICD-10 code F31	Public health records	DM (ICD-10 codes E10–E14)	Public health records	Risk of DM (1.7-fold among women and 1.6-fold among men)	>20	6587,036 353,615 with DM 6618 with BD
Bai et al.	2013	Risk of developing diabetes mellitus and hyperlipidemia among patients with bipolar disorder, major depressive disorder, and schizophrenia: a 10-year nationwide population-based prospective cohort study	10-year nationwide population-based prospective matched control cohort study	BD (ICD-9-CM code: 296, except 296.2, 296.3)	National Health Insurance (NHI) program records	DM (ICD-9-CM code 250)	National Health Insurance (NHI) program records	Increased risk of initiation of anti-diabetic medications (10.1 vs. 6.3 %, *p* = 0.012) Age and gender adjusted risk [hazard ratio (HR) of 1.702, 95 % confidence interval (CI): 1.155–2.507]	Average age 45.3 ± 14.0	1000,000 367 patients with BD 37 with DM
Svendal et al.	2012	Co-prescription of medication for bipolar disorder and diabetes mellitus: a nationwide population-based study with focus on gender differences	Norwegian prescription database Case–control study	BD	Indicated by prescription of mood stabilizers	DM	Indicated by prescription of antidiabetic medication	Unadjusted odds ratio of 2.1 (CI 95 %: 1.9, 2.2) Sex and age adjusted odds ratio of 2.0 (CI 95 %: 1.8, 2.1)	20–69	2,929,065 77,669 with DM 17,007 with BD
Hsieh et al.	2012	Medical costs and vasculo-metabolic comorbidities among patients with bipolar disorder in Taiwan—a population-based and -matched control study	Matched case–control study	BD (ICD-9-CM code 296, except 296.2, 296.3)	Hospital admission	DM ICD-9-CM (250)	Medical records	DM prevalence ratio 3.19; [2.74, 3.70]; p < .0001	>20	About 23,000,000 4,067 with BD, 420 with DM
Kodesh et al.	2012	Epidemiology and comorbidity of severe mental illnesses in the community: findings from a computerized mental health registry in a large Israeli health organization	Publicly funded Health Maintenance Organization (HMO) records Case–control study	BD-I, BD-II, Mania ICD-9 codes 295.*–298.*	Medical records	DM	Computerized medical records	DM odds ratio of 1.6	>21	2,000,000 5,732 patients with BD
Chien et al.	2010	Prevalence of diabetes in patients with bipolar disorder in Taiwan: a population-based national health insurance study	National Health Research Institute Case–control study	BD	Medical records	DM	Medical records	Diabetes prevalence in BD patients versus controls 10.77 vs. 5.57 %, OR 2.01; 99 % CI 1.64–2.48	>18	1,000,000 1,848 with BD

## Results

### Bipolar disorder and diabetes mellitus: is there a connection?

The results of the seven large population-based studies published between January 2009 and December 2015 provided strong evidence for a correlation between BD and diabetes mellitus (Table 1). When compared to the population background, odds ratios for diabetes mellitus in patient populations with BD were in the range of 1.7–3.2. Reciprocally, BD was more common among those with diabetes mellitus compared to the general population when adjusted for age and gender (Wändell et al. 2014). A nationwide, population-based longitudinal cohort study found that patients with BD, who had no diagnosis of diabetes mellitus at baseline, were more likely to begin anti-diabetic medications over the 10-year course of the study, even after controlling for gender, urbanization, and income (Bai et al. 2013). Across all ethnic and racial groups, females seem to have additional risk. Glucose and lipids were dysregulated at high rates in patients with BD, particularly in women over age 40 (Wysokinski et al. 2015), and obesity, a major risk factor for diabetes mellitus, was highly prevalent (Goldstein et al. 2011).

The results of these very large studies are consistent with the results of previous literature reviews covering smaller studies up to 2012, which found that diabetes mellitus occurs up to three times as often among individuals with BD, as it does in the general population (Calkin et al. 2013; Janssen et al. 2015). However, some studies also indicated that metabolic dysfunctions in patients with BD are frequently underdiagnosed (Carliner et al. 2014; Konz et al. 2014).

## Discussion

### Bipolar disorder and diabetes mellitus: do these disorders share common disease mechanisms?

The results of our study suggest a relationship between BD and diabetes mellitus. Therefore, we reviewed the supporting evidence for shared disease mechanisms based on the broader literature.

A common explanation for the association between BD and diabetes mellitus focuses on the diabetogenic side effects of psychotropic medications, but evidence is also increasing for a medication-independent association (Foley et al. 2015). While diabetes mellitus in patients with BD has been associated with unintended medication effects (Correll et al. 2015), antipsychotics are more strongly linked to incident diabetes mellitus than other treatments, such as mood stabilizers and antidepressants. Among the antipsychotics, olanzapine and clozapine (both second generation antipsychotics) have been most strongly linked to diabetes mellitus, because they block insulin secretion as antagonists of acetylcholine muscarinic 3 receptors in the β-cells of the pancreas (Thakurathi and Henderson 2012; Weston-Green et al. 2013). A sedentary lifestyle has been claimed as another contributing factor to the increased risk of diabetes mellitus in patients with BD (Perseghin et al. 1996; Gomes et al. 2013; Janney et al. 2014; Conn et al. 2014). However, even after accounting for antipsychotic exposure and lifestyle factors, the higher incidence of diabetes mellitus among patients with BD remains unexplained, especially in treatment-naïve patients (Lilliker 1980; Cassidy et al. 1999; Regenold et al. 2002; Ruzickova et al. 2003; McIntyre et al. 2005; Maina et al. 2008; García-Rizo et al. 2014; Guha et al. 2014).

The observed association between BD and diabetes mellitus has inspired several hypotheses about shared disease mechanisms (Calkin et al. 2013). While some researchers have focused on dysregulations of the purine metabolism as a common link between energy homeostasis and neuro-regulation (Salvadore et al. 2010), others have proposed elevated cortisol levels related to imbalances in the hypothalamic–pituitary–adrenal axis, which consequently could result in obesity and derailment of the glucose metabolism (McElroy et al. 2004). A few researchers have hypothesized that insulin resistance in adipose tissue could be mediated by abnormalities in thyroid hormone receptor signaling pathways and gene regulation. Imbalances in thyroid hormones have long been suspected to be causally related to BD (Iwen et al. 2013). A new disease model hypothesizes that thyroid hormone receptor-associated protein 3 (Thrap3) could activate a diabetogenic gene cascade in adipose cells through interaction with cyclin-dependent kinase 5 (CDK5) leading subsequently to the phosphorylation of peroxisome proliferator-activated receptor γ (PPARγ) at Ser273 (Choi et al. 2014). An extension of this model included sleep abnormalities, which are frequently found in patients with psychiatric disorders, as a contributing factor to the manifestation of diabetes mellitus (Li et al. 2013). While thyroid hormone abnormalities have been convincingly linked to BD (Bauer et al. 2014), a causal link between thyroid abnormalities, diabetes mellitus, and mood symptoms continues to be a focus of intense investigations in cell culture and animal models (Wang 2013).

Increased insulin resistance is commonly considered an intermediate phenotype to the manifestation of diabetes mellitus. In patients with BD, an alternative pathomechanism has been explored in the context of the metabolic syndrome, a combination of obesity, diabetes mellitus, dyslipidemia and hypertension. The metabolic syndrome is very common in the general population, but it occurs at even higher rates in patients with BD (Fagiolini et al. 2005). While insulin resistance was not increased in patients with BD and metabolic syndrome compared to age, gender, and body mass index (BMI)-matched controls, patients with BD had a reduced capacity to utilize fat as an energy source. This abnormality could predispose BD patients to exacerbated weight gain and increased risk for diabetes mellitus and cardiovascular disease (Fleet-Michaliszyn et al. 2008).

Perhaps the most intriguing hypothesis linking BD and diabetes mellitus has focused on underlying immune dysfunctions paired with a chronic inflammatory state, which could confer risk for both BD and diabetes mellitus (Leboyer et al. 2012; Hamdani et al. 2013; Sharma et al. 2014; Rosenblat and McIntyre 2015; Kim et al. 2015). This argument is supported by findings of increased susceptibility to allergies and elevated pre-inflammatory markers in BD and in diabetes mellitus (Goldstein et al. 2009; Wang et al. 2013; Chen et al. 2014). Oxidative stress could also lead to cell damage and apoptosis in the pancreas and in the brain, suggesting shared environmental risk factors for BD and diabetes mellitus (Reininghaus et al. 2014; Wright et al. 2006; Chang and Chuang 2010). This disease mechanism has been convincingly demonstrated in rat pancreatic β-cells, in which increased β-cell apoptosis was initiated by endoplasmic reticulum (ER) stress, mediated by abnormal glycogen synthase kinase-3β (GSK-3β) and caspase-3 activity. Valproic acid inhibited GSK-3β, which resulted in a cytoprotective effect. While this disease mechanism still awaits confirmation in patients with BD, the striking results suggest abnormal GSK-3β activity as a common link between BD and diabetes mellitus supported by a potentially similar drug effect of valproic acid on GSK-3β in the pancreas and in the brain (Huang et al. 2014).

### Bipolar disorder and diabetes mellitus: what are the outcomes?

The impacts of BD and diabetes mellitus on each other appear to be substantial. Recent work by Calkin et al. found that patients with BD and diabetes mellitus or insulin resistance had three times higher risk of having a chronic course of BD compared to euglycemic BD patients; patients with either type of insulin dysregulation also had three times higher risk of rapid cycling and were more likely to be refractory to lithium (Calkin et al. 2015). In a study of 82,060 patients with diabetes mellitus admitted to community hospitals over a 2-year period in Washington State, having a serious mental illness significantly increased the odds of rehospitalization for non-mental conditions within 1 month of discharge (odds ratio 1.24, 95 % confidence interval 1.07–1.44), even after controlling for demographics, medical co-morbidity, and index hospitalization (Chwastiak et al. 2014). Among the 2.2 % with comorbid serious mental illness, 60 % had a diagnosis of BD, which was consistent with previous studies (Callaghan and Khizar 2010). Other studies confirmed that diabetes mellitus increased hospital-based mortality in patients with BD (Schoepf and Heun 2014; Sylvia et al. 2015).

Worryingly, BD and diabetes mellitus are each independently associated with increased risk of dementia and reduced cognitive performance (Biessels et al. 2006; Xu et al. 2009; Wu et al. 2013; Zilkens et al. 2014; Depp et al. 2014). After controlling for vascular risk factors, patients with diabetes mellitus show increased evidence for global brain atrophy relative to age- and gender-matched controls (Wisse et al. 2014; Biessels and Reijmer 2014), including reduced gray matter density, reduced cerebral glucose metabolism in frontotemporal regions (García-Casares et al. 2014), increased ventricular volume (De Bresser et al. 2010), and white matter hyper-intensities (Reijmer et al. 2011). When compared to euglycemic BD patients and non-psychiatric controls, the BD patients with insulin resistance or glucose intolerance and diabetes mellitus had significantly more neurochemical changes in the prefrontal cortex, indicating reduced neuronal health (Hajek et al. 2015). In one study, patients with BD and diabetes mellitus or insulin resistance also had significantly smaller hippocampal and cortical volumes than either euglycemic BD patients or controls (Hajek et al. 2014).

Separately, each disease is associated with increased mortality. Diabetes mellitus is the seventh leading cause of death (Center for Disease Control 2014). Among adults 18 years and older during the years 2003–2006 in the US, a diagnosis of diabetes mellitus increased all-cause mortality about 1.5 times over non-diabetics. For BD, a Swedish national cohort study has shown that, relative to the general population, men and women with BD died on average 8.5 and 9.0 years earlier, respectively, and for each gender, having BD increased the risk of death by twofold (Crump et al. 2013). BD patients have a 20-fold greater risk of suicide relative to the general population (Jann 2014). Meanwhile, those with BD in addition to diabetes mellitus have increased mortality rates of 1.47 (95 % CI 1.07–2.02) versus those with diabetes mellitus but not BD (Vinogradova et al. 2010).

## Outlook

### Investigations into treatment implications

Both diabetes mellitus and BD are highly refractory: less than half of the participants in the National Health and Nutrition Examination Survey (NHANES) met glycemic control goals (Koro et al. 2004). BD patients in general have high rates of treatment non-adherence and recurrence. Furthermore, a strong association between HbA1c levels and symptoms of depression has been described in patients with BD (Bajor et al. 2015; Sajatovic et al. 2015b). Because of the difficulties in arresting progression of diabetes mellitus, achieving lifetime remission from BD, and the high stakes involved in both diseases, new treatment avenues, especially those that treat the potentially shared disease mechanisms of diabetes mellitus and BD, are desirable.

In the search for new drug targets, glycogen synthase 3 (GSK-3) has taken center stage for its known involvement in several pathways linked to both BD and diabetes mellitus (Gould et al. 2004; Ronai et al. 2014; Huang et al. 2014; Iwahashi et al. 2014). In the rat, lithium, a standard treatment for BD, reduces the enzyme’s activity in the hippocampus and improves memory and learning (Qu et al. 2014). Novel GSK-3 inhibitors are now in preclinical testing (Datusalia and Sharma 2014; King et al. 2013).

In addition to the GSK-3 pathway, dysregulation of noradrenaline signaling could potentially be a shared disease mechanism between BD and diabetes mellitus, which has led to investigations into prophylactic use of noradrenaline modulators (Fitzgerald 2015). With the intention to target inflammatory pathways, toll-like receptor (TLR)-modifying agents have been tried in diabetes mellitus and BD among others (Ladefoged et al. 2013; McKernan et al. 2011; Lucas and Maes 2013). Last, but not least, treatment with the antidiabetic drug pioglitazone as an adjunct to lithium improved symptoms of depression in patients with BD even in the absence of diabetes mellitus (Zeinoddini et al. 2015).

### Bipolar disorder in the elderly: does age of onset hint a distinct disease phenotype?

BD in the elderly poses specific challenges for diagnosis and treatment (Préville et al. 2008, 2010; Volkert et al. 2013; Sajatovic et al. 2015a). Although the usual gender ratio for BD is 1:1, in elderly patients, more women than men receive treatment for BD. Lower overall cognitive and executive functioning have been reported in older patients with BD compared to both younger patients and normal controls in some studies (Tsai et al. 2009; Sheeran et al. 2012). However, not all studies have supported these conclusions (Delaloye et al. 2011). Age of onset of BD might be a confounding factor.

While BD usually presents with an age of onset during adolescence and early adulthood, some individuals experience a first episode of mania in and beyond the 5th decade of life (Bellivier et al. 2001, 2003; Kennedy et al. 2005). Most studies on BD in the elderly have not distinguished between early-onset and late-onset cases, but the evidence for a separate subtype of BD distinguished by age of onset is growing, if complex. Late-onset mania appears to have a distinctive phenotype, pathophysiology, and risk factors (Leboyer et al. 2005; Vasudev and Thomas 2010; Sheeran et al. 2012; Schouws et al. 2009, 2012; Sajatovic et al. 2005; Sajatovic and Chen 2011; Sajatovic et al. 2015a). In several studies, the late-onset group differed in psychiatric comorbidities, including lower rates of lifetime alcohol and substance abuse, and lower rates of anxiety disorders. In some studies, elderly patients with late-onset BD performed particularly worse on tests of psychomotor function and mental flexibility compared to those with BD who had an earlier age of onset, though elderly patients with BD from both groups performed more poorly than age-matched controls (Schouws et al. 2009, 2012). An increasing burden of chronic health problems has been related to the risk of late-onset BD including diabetes mellitus, hyperlipidemia, and other cardiovascular conditions (Préville et al. 2010; Sylvia et al. 2015), whereas in BD individuals with younger age of onset the risk is much less.

Investigations into the relationship between BD and diabetes mellitus have generally focused on all ages of patients. Even though late-onset cases of BD were not explicitly excluded in most studies, we noticed that few studies clearly distinguished between early-onset and late-onset cases of BD. However, this distinction could be quite relevant to treatment and outcome. Reports that particularly focused on late-onset BD and diabetes mellitus were sparse, and large studies were non-existing.

### Gaps in knowledge and limitations of our study

Even though diabetes mellitus and BD in the elderly are growing public health problems, clinical studies on these topics are sparse. In general, available studies still suffer from methodological problems including small sample size, limitations of retrospective chart review, lack of standardized measures, overemphasis on inpatients, and lack of longitudinal data. Several studies have addressed not only the increasing healthcare utilization in elderly patients with BD and medical comorbidity, pointing to a need for integrated medical and psychiatric care in this vulnerable population (Hendrie et al. 2013), but also to existing healthcare disparities for patients with mental illness (Gierisch et al. 2014; McGinty et al. 2015).

In our literature review, we have been unable to identify published large-scale, multi-center studies on the prevalence, the etiology, or the clinical features of late-onset BD. To our knowledge, no double-blind, randomized, controlled trials of pharmacologic treatments have been performed in this specific patient population. Therefore, we recommend to increase emphasis on research in BD during the late stages of the disease, which could inform about the disease course and risk factors across the lifespan. It is hoped that this knowledge will not only assist in enhancing services and improving outcomes, but it might also lead to the discovery of potentially new pathophysiological pathways and risk factors for BD and diabetes mellitus, as well as to novel treatments and interventions.

## Conclusions and recommendations

Increasing evidence supports the association between BD and diabetes mellitus and suggests shared risk factors and disease mechanisms. This public health problem deserves focused attention, especially in the elderly, to improve diagnosis, treatment and outcome. A stronger integration of medical and psychiatric care could help prevent the negative effects of these co-occurring disorders on the long-term outcome of patients with BD. Therefore, we recommend to increase research efforts on late-life BD and diabetes mellitus to better understand the complex relationship that exists between these disorders. A better understanding of risk factors in BD and diabetes mellitus could lead to novel treatment approaches, early intervention and prevention.

## Abbreviations

BD: bipolar disorder
DSM-IV: Diagnostic and Statistical Manual of Mental Disorders, 4th Edition
ICD: International Classification of Diseases
PPARγ: peroxisome proliferator-activated receptor γ
CDK5: cyclin-dependent kinase 5
Thrap3: thyroid hormone receptor-associated protein 3
ER: endoplasmatic reticulum
GSK-3β: glycogen synthase kinase-3β
NHANES: National Health and Nutrition Examination Survey
TLR: toll-like receptor
